# The improvement of functional handwashing facilities during COVID-19: the perspective of Tanzania

**DOI:** 10.1186/s41182-021-00334-y

**Published:** 2021-05-27

**Authors:** Magdalena Shao, Vivian Mushi, Luco Mwelange, Anyitike Mwakitalima

**Affiliations:** 1 Department of Environmental and Occupational Health, School of Public Health and Social Sciences, Muhimbili University of Health and Allied Sciences, P.O. Box 65001, Dar es Salaam, Tanzania; 2 Department of Parasitology and Medical Entomology, School of Public Health and Social Sciences, Muhimbili University of Health and Allied Sciences, P. O. Box 65011, Dar es Salaam, Tanzania; 3 Ministry of Health, Community Development, Gender, Elderly and Children, P. O. Box 573, Dodoma, Tanzania

**Keywords:** COVID-19, WASH, Handwashing with soap and water, Tanzania

## Abstract

Fighting against coronavirus disease-2019 (COVID-19) goes hand in hand with the provision of proper water, sanitation, and hygiene (WASH) services. In this case, proper handwashing with soap and water plays a major role in the prevention of COVID-19, since it helps to deactivate and remove virus particles from the hands. This letter points out the drivers for the improvement of functional handwashing facilities during the COVID-19 pandemic in Tanzania, whereby three out of every 20 non-functional handwashing facilities were improved to make them functional. The letter also provides several recommendations to maintain momentum for improving functional handwashing facilities.

To the Editor:

Severe acute respiratory syndrome corona virus 2 (SARS-CoV-2) has been identified as the causative agent for the COVID-19 outbreak. It is believed that SARS-CoV-2 has a zoonotic origin and transmission to humans was either from bats or through an unknown intermediate host [1, 2]. The spread of COVID-19 from human to human was possible via respiratory droplets or contact routes [3, 4]. As of April 22, 2021, COVID-19 had spread throughout the world, with more than 144,603,219 cases, and led to 3,075,168 deaths [5]. The World Health Organization (WHO) recommended handwashing with soap and water or an alcohol-based sanitizer as the best way to prevent further spread of the virus [6]. However, it has been estimated that 3 billion people globally lack handwashing facilities at home and face the challenges related to inadequate water and knowledge of proper hand hygiene practices [7].

Tanzania is among the countries that took initiatives to prevent the spread of COVID-19 by adhering to WHO recommendations. Prior to the outbreak, 59% of Tanzanian households had handwashing facilities that included soap and water; however, only 22.83% of these facilities were functional [8, 9]. Therefore, the country implemented several measures beginning in March 2020. First, a massive campaign was launched through the National Sanitation Campaign with the aim of promoting the importance of handwashing with soap and water. The campaign was organized, coordinated, and implemented throughout the country by the Ministry of Health, Community Development, Gender, Elderly and Children. This campaign differed from previous efforts, in which programs were normally conducted based on normal, or typical, access to hardware and services (e.g., soap, washing facilities, and a water supply), but only limited promotion and an incomplete enabling environment. During the COVID-19 outbreak, there were highly prominent promotions of handwashing involving political leaders, including the country’s president, religious leaders, influential people, the media, and the collaboration of many stakeholders. Second, the Ministry of Water ensured a reliable supply of adequate water for the general public. Moreover, the government, working in collaboration with development partners, provided an enabling environment and installed handwashing facilities in public areas. The initiative involved the distribution of hand sanitizers in those public areas that had continued to provide services, such as police stations, health care facilities, and other public offices.

According to data from the Ministry of Health, Community Development, Gender, Elderly and Children, which can be accessed through the Tanzania WASH portal, the coverage of the functional handwashing facilities has always been low, but it is also characterized by a gradual increase over time. As the above initiatives were implemented during the onset of COVID-19 in Tanzania, however, there was a rise in the trajectory of functional handwashing facilities, in terms of percentage increase. This is because the implemented measures resulted in a 14.81% increase in the number of households with functional handwashing facilities, from 22.83% in September 2019 to 37.64% by June 2020 [9].

It can be concluded that, for every 20 non-functional handwashing facilities in the country, three were made functional through the various measures discussed above. This is a good start that should be improved upon and maintained, because it will help in the fight against COVID-19 and other infectious diseases. We must insist on the provision of adequate handwashing facilities, an enabling environment, and the promotion of proper hand hygiene, to ensure that Tanzanians achieve sustainable handwashing behaviors throughout the country.

We recommend the following to maintain this momentum. There should be a show of political will and good leadership. This was observed during the onset of COVID-19, when many political leaders, including the president, participated in initiatives to raise awareness of the importance of hand hygiene. The involvement of these key figures made the campaign a success. The government should increase the amount of resources allocated to hygiene issues (of which handwashing is part and parcel) and water supply, to make proper handwashing practices possible.

Quick and concerted actions should be taken all over the country to make sure awareness is raised to the level that allows the country to attain 100% coverage as far as handwashing facilities are concerned, since this is the first line of defense in the prevention of infectious diseases.

Handwashing facilities should be easily accessible to all, so as to convince more people to acquire and use them. It is important to ensure that handwashing facilities are available in all public areas, such as markets, stadium, bus terminals, and in all schools and colleges. The provision of handwashing facilities at entry points should be mandatory in all health care facilities.

The focus should not only be on improving WASH infrastructures, but also on behavioral change. Proper handwashing behaviors should be encouraged even after the COVID-19 pandemic, because it will be useful for controlling other infectious diseases. The country’s health policy should also be revised to include a WASH component in times of pandemics. This component should include a plan to ensure the availability, accessibility, and sustainability of WASH services in health care facilities, schools, households, and all public places.

## Data Availability

All the data used are from the references provided.

## Abbreviations

COVID-19: Coronavirus disease-2019
SARS-CoV-2: Severe acute respiratory syndrome corona virus 2
WHO: World Health Organization
